# Yellow fever outbreak in a rural-urban mixed community of Espírito Santo, Brazil: epidemiological aspects

**DOI:** 10.26633/RPSP.2019.29

**Published:** 2019-03-15

**Authors:** Tálib Moysés Moussallem, Caroline Gava, Karla Spandl Ardisson, Clemilda Soares Marques, Giselle Calmon Graceli, Aline da Penha Valadares Koski, Gilton Luiz Almada, Alexandre Rodrigues da Silva, Fernando Antonio Alves de Jesus, Gilsa Aparecida Pimenta Rodrigues, Theresa Cristina Cardoso da Silva

**Affiliations:** 1 Espírito Santo State Health Secretariat Espírito Santo State Health Secretariat Special Center for Epidemiological Surveillance VitóriaEspírito Santo Brazil Special Center for Epidemiological Surveillance, Espírito Santo State Health Secretariat, Vitória, Espírito Santo, Brazil.; 2 Oswaldo Cruz Foundation Oswaldo Cruz Foundation Sergio Arouca National School of Public Health Rio de JaneiroRio de Janeiro Brazil Sergio Arouca National School of Public Health, Oswaldo Cruz Foundation, Rio de Janeiro, Rio de Janeiro, Brazil.; 3 Espírito Santo State Health Secretariat Espírito Santo State Health Secretariat Center for Strategic Information and Responses in Health Surveillance VitóriaEspírito Santo Brazil Center for Strategic Information and Responses in Health Surveillance, Espírito Santo State Health Secretariat, Vitória, Espírito Santo, Brazil.; 4 Espírito Santo State Health Secretariat Espírito Santo State Health Secretariat Dório Silva Hospital SerraEspírito Santo Brazil Dório Silva Hospital, Espírito Santo State Health Secretariat, Serra, Espírito Santo, Brazil.; 5 Espírito Santo State Health Secretariat Espírito Santo State Health Secretariat Health Surveillance Management VitóriaEspírito Santo Brazil Health Surveillance Management, Espírito Santo State Health Secretariat, Vitória, Espírito Santo, Brazil.

**Keywords:** Yellow fever, disease outbreaks, mass vaccination, Brazil, Fiebre amarilla, brotes de enfermedades, vacunación masiva, Brasil, Febre amarela, surtos de doenças, vacinação em massa, Brasil

## Abstract

**Objective.:**

To describe the epidemiological aspects of an outbreak of yellow fever (YF) that occurred in the state of Espírito Santo, Brazil, from 1 January 2017 – 31 July 2017.

**Methods.:**

A descriptive, quantitative, retrospective approach analyzed secondary data obtained from the national notification systems, Information System of Diseases Notifications (SINAN), Laboratory Environment Manager (GAL), and the Espírito Santo Health Secretariat (SESA).

**Results.:**

From 1 January 2017 – 8 July 2017, a total of 824 cases were reported in Espírito Santo, 307 (37%) of which were confirmed as YF. Of these, 95 (30.9%) died from the disease. Men were those most affected, corresponding to 244 (79.5%) cases, and women to 63 (20.5%) cases. The greatest incidence rate registered was in the city of Santa Leopoldina (380.2 cases/100 000 inhabitants). The outbreak evolved rapidly and a response was possible due to a multidisciplinary group created specifically to tackle the YF outbreak.

**Conclusions.:**

The data were received and analyzed quickly and the response, consisting of immediate treatment of the cases and a blocking vaccination strategy, was developed to halt the progression of this fatal disease. In spite of these efforts, the case fatality rate of yellow fever remained high.

## RESUMEN

**Objetivo.:**

Describir los aspectos epidemiológicos de un brote de fiebre amarilla ocurrido en el estado de Espírito Santo (Brasil), del 1 de enero al 31 de julio del 2017.

**Métodos.:**

Por medio de un enfoque descriptivo, cuantitativo y retrospectivo se analizaron los datos secundarios obtenidos a partir de los sistemas de notificación nacionales, el sistema informático de notificación de enfermedades (SINAN), el sistema de gestión del entorno de laboratorio (GAL) y la Secretaría de Salud de Espírito Santo (SESA).

**Resultados.:**

Del 1 de enero al 8 de julio de 2017 se notificó un total de 824 casos de fiebre amarilla en Espírito Santo, de los cuales 307 (37%) fueron confirmados. De estos, 95 (30,9%) murieron por causa de la enfermedad. El mayor número de afectados correspondió a la población masculina, con 244 casos (79,5%), mientras que 63 casos (20,5%) fueron mujeres. La tasa de incidencia más alta registrada se observó en la ciudad de Santa Leopoldina (380,2 casos/100 000 habitantes). El brote evolucionó rápidamente y se logró darle respuesta gracias a un grupo multidisciplinario formado específicamente para contrarrestar este brote de fiebre amarilla.

**Conclusiones.:**

Los datos se recibieron y analizaron rápidamente. La respuesta para detener la progresión de esta enfermedad mortal consistió en un tratamiento inmediato de los casos y una estrategia de vacunación de bloqueo. A pesar de estos esfuerzos, la tasa de letalidad de la fiebre amarilla continuó siendo alta.

## RESUMO

**Objetivo.:**

Descrever os aspectos epidemiológicos de um surto de febre amarela ocorrido no Estado de Espírito Santo, Brasil, no período de 1^o^ de janeiro de 2017 a 31 de julho de 2017.

**Métodos.:**

O estudo se baseou em um enfoque descritivo, quantitativo e retrospectivo para analisar dados secundários obtidos dos sistemas nacionais de notificação: Sistema de Informação de Agravos de Notificação (SINAN), sistema Gerenciador de Ambiente Laboratorial (GAL) e Secretaria de Estado da Saúde de Espírito Santo (SESA).

**Resultados.:**

No período de 1^o^ de janeiro de 2017 a 8 de julho de 2017, 824 casos foram notificados no Espírito Santo, sendo 307 (37%) confirmados como febre amarela. Ocorreram 95 casos de morte pela doença (30,9%). O sexo masculino foi o mais afetado, sendo registrados 244 casos (79,5%) no sexo masculino e 63 casos (20,5%) no sexo feminino. A taxa de incidência mais alta foi verificada na cidade de Santa Leopoldina, com 380,2 casos por 100 mil habitantes. O surto progrediu rapidamente e a resposta foi possível com a ação de um grupo multidisciplinar formado para combater o surto de febre amarela.

**Conclusões.:**

Os dados foram obtidos e analisados com rapidez e a resposta, consistindo de tratamento imediato dos casos e uma estratégia de vacinação de bloqueio, visou deter a progressão desta doença fatal. Apesar dos esforços, a taxa de letalidade da febre amarela continuou alta.

Yellow Fever (YF) is an acute arbovirus disease caused by YF virus, the prototype of the *Flaviviridae* family endemic to Africa and South America. It affects humans and nonhuman primates (NHP), which acquire the infection by the bite of infected mosquitoes. The disease has a wide range of presentations, ranging from asymptomatic cases (the vast majority) to devastating hemorrhagic fever, leading to death (1 – 4). Mild and moderate cases represent 20% – 30% of the symptomatic form of the disease, 10% – 20% are severe forms, and 5% – 10% have a malignant presentation (1, 4).

Yellow fever has two main cycles of transmission. The sylvatic (jungle) cycle (JYF), where the vectors are the *haemagogus* and *sabethes* mosquitoes; and the urban cycle (UYF) caused by mosquitoes of the *Aedes* genus (3, 5, 6). There is another type of cycle, called the “intermediate cycle“described in the African savannah, which results in small scale epidemics in rural villages (5 – 7).

## Epidemiology of YF in Brazil

In Brazil, there has been no record of the YF virus being transmitted by the urban cycle since 1942. However, the circulation of the YF virus in the wild cycle is endemic in the Amazon area, with some outbreaks also occurring beyond that geographical area (9 – 13).

Espírito Santo is divided into 78 municipalities. The estimated population is 3 973 697 inhabitants, of which 16.5% live in rural areas (14 – 16). In December 2016, an outbreak of YF began in the state of Minas Gerais, which borders the state of Espírito Santo, in southeastern Brazil (14). Transmission of the disease spread to Espírito Santo, previously considered YF-free. Espírito Santo shares forested areas with Minas Gerais, where the wild vectors (*haemagogus* and *sabethes* mosquitos) are known to be present, but YF vaccine was not among the recommended vaccinations.

In 2003, a temporary recommendation for vaccination against YF had been issued for northern Espírito Santo due to a potential risk for YF transmission (13). However, the vast majority of the population was not immunized, making Espírito Santo a major scenario for disease transmission and resulting in the 2017 epidemic.

This study analyzes the epidemiological aspects of the wild YF epidemic in Espírito Santo in 2017, identifying the groups most susceptible to infection and describing control measures taken by the state government.

## MATERIALS AND METHODS

This was a descriptive, quantitative, retrospective study, using secondary data from notifications and research reports sent to the Espírito Santo Emergency Operations Center for Public Health (COES-ES) available in Microsoft Excel™ (Microsoft Corp., Redmond, Washington, United States) database files, as well as laboratory results available in the GAL. The Excel files, titled “Monitoring of Suspected Human Cases of YF from Espírito Santo” and “Monitoring of Epizootics Suspected of YF in Espírito Santo,” tracked YF data from 1 January 2017 – 8 July 2017 (Epidemiological Weeks 01 – 27).

### Data analyses

The analyses included only those cases for which Espírito Santo was the Probable Place of Infection (PPI). The description of case distribution, calculation of incidence rate, and fatality were determined according to municipality of PPI and epizootics by municipality of notification/occurrence. The incidence in the denominator of the estimated 2016 population for each municipality (15) per 100 000 population was used for calculating incidence. Laboratory results and vaccine coverage by municipality were also described. The maps were drawn by using the QGIS 3.0.3 program (Free and Open Source Geographic Information System, https://www.qgis.org/en/site/).

### Case definition and diagnosis

Given the epidemiologic situation of Espírito Santo in 2017, COES-ES amplified the case definition of a YF suspected case and created a new one to capture all presentations of the disease: “Patient with abrupt-onset fever (up to 7 days of duration), whether or not that is followed by jaundice and/or bleeding manifestations, living in or coming from risk areas for yellow fever or from places with confirmed epizootic cases in nonhuman primates or with viral isolation in mosquito vectors in the last 15 days, who have not been vaccinated against yellow fever or have unknown vaccination status” (17, 18).

The diagnosis of YF virus infection was done using several methods. For human blood samples, reverse transcription polymerase chain reaction (RT-PCR) and viral isolation techniques were used until the 5th day of infection following symptom onset, after which enzyme-linked immunosorbent assay (ELISA) was used. In fatalities, the same techniques were used on blood samples, and immunohistochemistry was added for samples of viscera obtained from autopsies.

The Central Laboratory of Espírito Santo (LACEN-ES) in Vitória, Espírito Santo, Brazil, performed the virus isolation and the serology tests. Human RT-PCR was performed by the Virology Laboratory of the Oswaldo Cruz Foundation (FIOCRUZ; Rio de Janeiro, Brazil) on material sent by LACEN-ES. Immunohistochemistry to confirm YF was performed at the reference laboratory of the Evandro Chagas Institute in Belém, Pará, Brazil.

Samples from nonhuman primates were sent to the Evandro Chagas Institute (Belém, Pará, Brazil).

#### Ethics.

This study was carried out with the authorization of SESA through the Research Request Process Flowchart in accordance with the rules in force in Administrative Rule 040/ES/2015. To preserve the anonymity of the data, the patients were identified by initials only in the statistical analysis.

## RESULTS

From 1 January 2017 – 8 July 2017, a total of 824 suspected human YF cases with PPI in Espírito Santo and 882 NHP cases also with PPI in Espírito Santo, were reported to COES-ES. Of the 824 reported human cases, 307 (37%) were confirmed—274 by laboratorial diagnostic tests. In January 2017, the first suspected YF human cases in Espírito Santo were along the border with Minas Gerais, in the municipalities of the southwest (Ibatiba) and northwest (São Roque do Canaã and Colatina).

**FIGURE 1:**
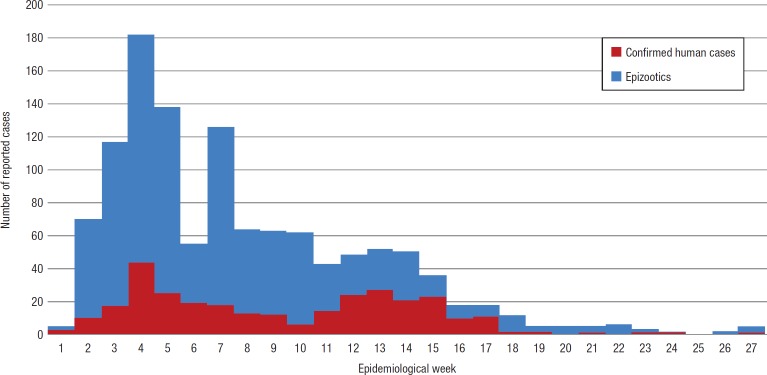
Distribution of human and epizootics yellow fever cases, by epidemiological week, Espírito Santo State, Brazil, 1 January – 8 July 2017

***Source:*** Prepared by the authors from the study results.

From the beginning of the outbreak, the number of cases rose progressively, peaking by epidemiologic week (EW) 04 (22 – 28 January 2017) for human and NHP cases. EW 04 saw an increase of 144% in human cases over the previous week (Figure 1); among epizootics, 40%. Human cases then declined until EW 10 (5 – 11 March 2017), followed by a new “wave” of cases in EW 11 that peaked at EW 13 (26 March – 1 April 2017). Reductions then followed as of EW 16, being more expressive in EW 18 (30 April – 6 May 2017). Among the NHPs, a significant decline was noticed in week 06 (the period corresponding to the Carnaval holiday); however, the sustained reduction of epizootics occurred as of EW 08, being more expressive as of EW 16 (Figure 1).

### Incidence rates

**FIGURE 2:**
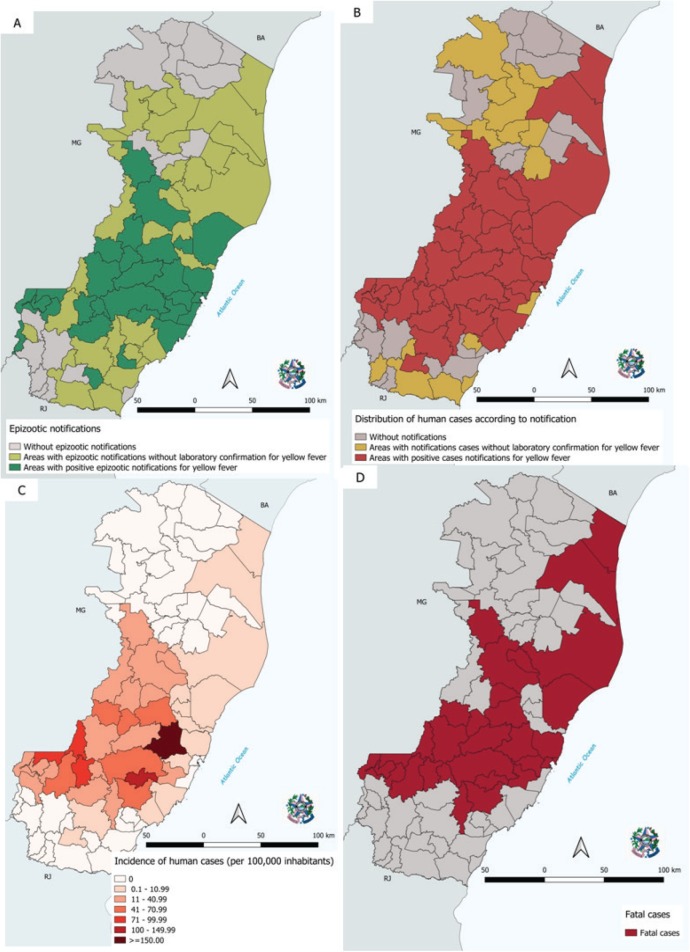
Spatial distribution of Yellow Fever cases in Espírito Santo, Brazil, according to epizootic notifications (A), human cases (B), incidence of human cases (C), and fatal human cases (D) from 1 January – 31 July 2017

***Source:*** Prepared by the authors from the study results.

It was observed that in all municipalities with confirmed human cases (except two municipalities in the south), there were occurrences of epizootics (Figures 2-A and 2-B). The incidence of YF human cases in Espírito Santo was 7.7 cases per 100 000 inhabitants, but some areas were more affected by the outbreak, leading to a heterogeneous distribution (Figure 2-C). For example, the municipalities of Santa Leopoldina and Marechal Floriano registered incidence rates of 380.2 and 110.2 cases per 100 000 inhabitants, respectively; whereas neighboring municipalities Domingos Martins, Santa Teresa, and Santa Maria de Jetibá showed incidence rates of 63.6, 54.4, and 30.5 cases per 100 000 inhabitants, respectively.

### Demographics

The median age of the YF patients was 43.9 years, ranging from 2 – 83 years (Table 1). Regarding gender, men were those most affected, accounting for 79.5% of the confirmed cases (*n* = 244; median age 42.8 years, range 2 – 83 years), with the greatest concentration in those 40 – 49 years (25.0%). Women were less affected by the outbreak (*n* = 63; median age 48.3 years, range 11 – 82 years) and the greatest concentration of cases was in those 60 – 69 years of age (28.6%).

People working in rural areas (farm laborers, farmers, and cattlemen) represented 50.8% of the cases.

### Fatality rates

There were 95 confirmed YF-related deaths. Those most affected were 40 – 49 years of age (25.3%; *n* = 24; median age 46.6 years, range 11 – 83 years); men (85.3%; *n* = 81) and rural occupations (55.8%; *n* = 53) predominated. With regards to municipalities with 10 or more registered cases, the greatest case fatality rates were found in Santa Maria de Jetibá (66.7%) and Muniz Freire (60.0%). The general fatality rate in Espírito Santo was 30.9%.

### Diagnostics

Regarding the diagnostic tests of human cases, LACEN-ES isolated the YF virus in 23.9% (*n* = 73) of the samples. Isolation of the virus occurred in samples from 25 different municipalities. Positivity, determined by RT-PCR, occurred in 63.2% (*n* = 194) of samples analyzed. Only 10.8% (*n* = 33) of the human cases were confirmed by clinical-epidemiologic criteria.

Of the 389 NHP samples sent to the reference laboratories (Oswaldo Cruz Foundation, Evandro Chagas Institute, and Adolfo Lutz Institute), 167 (43%) were analyzed for immunohistochemistry and molecular biology. In 71.3% (*n* = 119), there was confirmation of YF samples from 28 municipalities (Figure 2). The genus of 421 (31.6%) monkeys was identified: Alouatta (54.6%), Callithrix (42.0%), and Cebus (3.4%).

## DISCUSSION

The present study describes the largest outbreak of JYF ever registered in Brazil. The highest incidence rates occurred in the state of Espírito Santo, most cases in the highlands area where the native forests are protected. Virus activity was probably intense deep within the forests for several months prior to the outbreak. When the virus reached the edges of the vegetation, where cities and forests mix, contact between humans and the haemagogus vector increased significantly, especially among individuals with forest-related occupations.

Espírito Santo was considered a “YF-free” area until the 2017 outbreak. The magnitude of this outbreak surpassed that of the outbreak of 1930, the worst on record (11). In the interim, some sporadic outbreaks had been reported outside the Amazon region, but none of such severity (9, 10, 19, 20).

The absence of sufficient notification in northern Espírito Santo seems to be partly at fault for the outbreak, seeing as the state of Bahia, which borders Espírito Santo to the north, reported cases of YF epizootic and human disease in its southern region. Another explanation for the absence of notifications was the mass immunization campaign in 2003, when the vaccination area was expanded to include this part of the state and its population became immune to the disease (13).

Case distribution per EW in Espírito Santo corresponded to the seasonal period of the disease in the country (10); and during the wet season, more men than women were affected, as previously described in the literature (1). The most affected age group was comprised of individuals 30 – 59 years of age—a concentration of 3/5 of the confirmed cases, most linked to exposure through rural activities. Rural activities/occupations accounted for more than one-half of the confirmed cases. Outbreaks in other states reported higher incidence rates among younger age groups than in Espírito Santo (1, 10, 12). Women were most affected at older ages, probably because older women work at rural activities, while younger women are more frequently involved in activities typical of urban areas, reducing their exposure.

**TABLE 1:** Yellow fever case distribution and incidence by age, Espírito Santo State, Brazil, 1 January – 8 July 2017

Age (years)	Number of cases	Incidence/100 000
1 – 9	8	0.2
10 – 19	20	0.5
20 – 29	36	0.9
30 – 39	51	1.3
40 – 49	70	1.8
50 – 59	60	1.5
60 – 69	51	1.3
70 – 79	9	0.2
> 80	2	0.1

***Source:*** Prepared by the authors from the study results.

The YF fatality rate in Espírito Santo was slightly lower than that recorded by other regions of Brazil (10, 21) during the 2017 outbreak and was lower than that of other occurrences described elsewhere (1, 7, 10, 13, 14). The lower fatality rate could be the result of the reference network created by the COES-ES and the Espírito Santo urgent and emergency care departments for severe cases.

In January 2017, the COES-ES was structured to develop YF surveillance and control strategies. A multidisciplinary team consolidated all notifications of suspected human and epizootic cases in Espírito Santo, evaluating and qualifying them daily, and reporting results to other agencies and secretariats so that they might act.

Another factor in the lower fatality rate was the detection of mild and moderate cases. The new case definition allowed reporting of suspected cases that did not present with jaundice and hemorrhage—only 1/3 of cases exhibited this classic form of the disease. It is important to underscore the relevance of laboratory diagnosis right from the start of the outbreak. This surveillance service focuses on differentiating YF from other arboviruses in circulation whose mild cases have similar clinical presentations (dengue, Zika, and chikungunya). It should be reiterated that performing viral isolation enabled better use of samples, as well as the use of RT-PCR for YF, which might have amplified the power of the laboratory response and confirmation of 2/3 of the total cases.

The YF vaccine has been used in Brazil since 1937 and is recommended for everyone 9 months of age and older living in and/or traveling to risk areas (3, 7, 8, 13, 19, 20). Espírito Santo has never had compulsory YF vaccination, except for a few cases of travelers and people living in the northern area previously described. The state’s population was largely not immunized against the virus.

A serious situation developed in Espírito Santo when a non-immune population was exposed to intense virus activity in areas where forests and cities mix. To confront the threat, the state government adopted strategies to rapidly immunize the population against YF, first targeting the municipalities with evidence of viral circulation and their neighborhoods (expanded areas), repeating the strategy previously used (10, 21). These areas were progressively broadened to include the entire state. Despite efforts at containment vaccination, the protective effects of YF vaccine take 10 days to develop. Given the incubation period of the disease, several vaccinated individuals developed yellow fever during this “gap” period.

The epidemic may have been exacerbated by the public’s lack of credence regarding the disease’s spread and deadliness. The municipality of Santa Leopoldina was a classic example of the public underestimating yellow fever. A significant part of the population did not get vaccinated despite government warnings of the outbreak’s explosive spread from the west. This municipality was the one most responsible for the second peak of the outbreak in Espírito Santo.

Given the aforementioned, it can be affirmed that implementing a YF vaccination campaign in high risk areas, such as near the state lines, was not ideal since the spread of viral circulation was faster than the immunological effect of the vaccine. However, mass vaccination and actively searching for the disease, especially in rural areas, certainly prevented a more drastic outcome.

Also, worth noting is that this outbreak could not be classified as UYF because *A. aegypti* was not the vector; only the haemagogus genus was found. The majority of cases could be easily classified as JYF; but some cases from areas near the coast (where the forest is sparse) were extremely difficult to define as jungle or urban. This led the researchers to surmise that something happened in Espírito Santo similar to the “intermediate” cycle previously described (15, 20). In non-published reports, some researchers have put forth the hypothesis that *A. albopictus* might possibly be the vector responsible for an intermediate cycle in South America.

Furthermore, the YF outbreak revealed its great potential to spread via uncontrolled epizootic cases. NHPs are extremely susceptible to YF virus and its spread among them cannot be stopped. Although 882 were reported to COES-ES, it is estimated that many more NHPs were affected. Many specimens could not be evaluated due to difficulty accessing their habitat. The advanced state of putrefaction of several specimens was also a challenge. It is known that epizootics are a warning sign for the risk of YF transmission to humans, i.e., the disease in non-human primates usually precedes occurrence of human cases (10, 22).

A significant concentration of epizootics was observed in Espírito Santo in January 2017 when human cases were already occurring. This led to the suspicion that epizootics were already occurring on a large scale, but had not been detected by surveillance services. Figure 1 shows a gap in epizootic reports for EW 6, which can possibly be attributed to Carnaval, a week-long holiday during which many reporting units operate with a skeletal crew only. As with other outbreaks in Brazil (1, 3, 22), the genus Alouatta was the most affected among NHPs. The first municipalities affected in Espírito Santo were the ones neighboring Minas Gerais, from which the epizootic cases came.

Although a large-scale vaccination strategy against YF has been adopted across the state (starting in primarily-affected areas and extending to coastal areas), seroconversion occurs in at least 10 days (3, 8, 21). Given its incubation period, the disease can develop during this gap period. It is also important to note that YF was transmitted to humans with some vaccine resistance, more so among the elderly, despite the state government’s campaigns and publicity, including house-to-house canvassing to urge the public to get vaccinated. It is important to point out that for many YF cases confirmed in Espírito Santo, the PPI was urban areas and the outskirts of cities, including the coastal region (in the metropolitan part of the state). Since Espírito Santo has a formidable presence of urban vectors (*Aedes*), a favorable scenario was established for re-urbanization of the YF virus transmission cycle, as discussed by other authors (7, 21, 23). The researchers believe that vaccination prevented the potential outcome.

### Limitations.

This study has some limitations, including the use of secondary data (not directly acquired by the authors) and a lack of details regarding the epizootics that certainly preceded the human epidemic.

## Conclusions

This is the first description of a yellow fever outbreak of such proportions in southeastern Brazil, and almost certainly the first in the Americas to include the “intermediate” transmission cycle of the disease.

The 2017 epidemic of YF in Espírito Santo occurred predominantly in areas where humans come into close contact with forests, mainly rural areas. Those most affected were adult working men and rural workers and, unlike in other studies, this epidemic mostly affected older age groups. Urgently instituting a specific multidisciplinary group composed of several aspects of YF surveillance and control—environmental, entomological, epidemiologic, laboratory, immunization, and health care—provided greater and earlier case detection and more timely intervention.

Future studies should be performed in metropolitan areas and city outskirts to improve the definition of the vectors involved in YF transmission at an intermediate cycle, neither jungle nor urban. Now designated as an area of risk, YF vaccination must be maintained indefinitely in Espírito Santo; likewise, YF should be included on the state’s list of differential diagnoses of acute febrile diseases.
